# Risk of adverse pregnancy outcomes and seroprevalence for brucellosis in pregnant women exposed to goats or raw goat products in southern Thailand: a prospective cohort study

**DOI:** 10.1186/s12884-019-2267-x

**Published:** 2019-04-05

**Authors:** Kan Kledmanee, Tippawan Liabsuetrakul, Somporn Sretrirutchai

**Affiliations:** 1 Epidemiology Unit, Faculty of Medicine, Prince of Songkla University, Songkhla, Thailand; 2 Department of Pathology, Faculty of Medicine, Prince of Songkla University, Songkhla, Thailand

**Keywords:** Adverse pregnancy outcome, Brucellosis, Exposure, Goat, Pregnant women, Raw goat products

## Abstract

**Background:**

Pregnant women infected with brucellosis have been shown to have higher odds of having been exposed to goats and raw goat products and adverse pregnancy outcomes. However, information on these associations in asymptomatic pregnant women is limited, particularly in the brucellosis-endemic areas. This study aimed to assess the association of a history of exposure to goats and/or raw goat products and the serological status of anti-*Brucella abortus* immunoglobulin G (IgG) with adverse pregnancy outcomes among pregnant women, and explore factors associated with having exposure to goats and/or raw goat products.

**Methods:**

A prospective cohort study was conducted among pregnant women from July 2015 to July 2016 at Songkhla province in southern Thailand. All pregnant women who came for antenatal care (ANC) visits were approached. Blood samples from the women who agreed to participate were randomly tested for anti-*Brucella abortus* IgG. The women were then followed for adverse pregnancy outcomes.

**Results:**

Of 666 pregnant women, the majority (74.4%) were aged 20–34 years and Muslim (89.2%), 30.6% indicated exposure to goats or raw goat products, and 17.3% had adverse pregnancy outcomes. Women rearing goats at home or having neighbors rearing goats were more likely to be exposed to goats or raw goat products by cutaneous contact. Of 465 women having a blood test, 3.7% had seropositive results for anti-*Brucella abortus* IgG. No association with adverse pregnancy outcomes was found in the women reporting any exposure to goat and raw goat products. Having the first ANC visit at the first trimester and history of preterm birth or low birth weight newborn were independent risk factors of adverse pregnancy outcomes. Women who had positive serological results were more likely to have a history of drinking raw goat milk than those who had negative results significantly.

**Conclusions:**

Although no association between past exposure with goats and raw goat products and adverse pregnancy outcomes was found, women with past exposure showed positive anti-*Brucella abortus* IgG. Counseling on avoiding consumption of raw goat milk would be beneficial to prevent goat-related infection in pregnant women in this area.

## Background

Adverse pregnancy outcomes, including abortion, stillbirth, preterm birth, and low birth weight, are common obstetric conditions indicating the quality of maternal and child services worldwide [1, 2]. Risk factors associated with these adverse outcomes through various mechanisms include maternal age, parity, body mass index, nutrient intake, stress and infections [3]. One of the risk factors for certain types of infections is various common pathogens being passed from infected animals to pregnant women, including *Listeria monocytogenes*, *Coxiella burnetti, Brucella melitensis, Brucella abortus, Chlamydia trachomatis, Chlamydia abortus,* and *Chlamydia pneumoniae* [4–7]. A study from Egypt showed significantly higher incidences of abortion (27% vs 15%) and intrauterine fetal death (13% vs 4%) in women with a positive titer of brucellosis compared to women with a negative titer [8]. Two in vitro studies showed that *Brucella* replicate in human trophoblasts and interfere with invasive capacity of extravillous trophoblast-like cells and produce proinflammatory responses which may contribute to pregnancy complications [9, 10].

Excretions from infected animals through urine, feces, and milk can contain zoonotic infectious agents which can be accidentally inhaled, ingested, or passed through direct contact via the skin. Many daily or occupational activities can involve potential pathogens as biological risks, either at home or through animal husbandry [11, 12]. For pregnant women, contact with animals, handling raw animal products, and consumption of undercooked meat or unpasteurized dairy products have been shown to be risk factors for adverse pregnancy outcomes among those living in zoonosis endemic areas [8, 11, 13, 14].

Brucellosis, caused by *Brucella* sp., is a zoonotic infection which can affect human reproductive health. The clinical manifestations of brucellosis can involve many systems and nonspecific manifestations such as fever, sweating, anorexia, weight loss, headache, fatigue, malaise, arthralgia and back pain [15]. It has been associated with spontaneous abortion, fetal death, preterm birth, and low birth weight [8, 16, 17]. Various studies have reported the seroprevalence of brucellosis among pregnant women with a history of adverse pregnancy outcomes from 1.8 to 25.0% [18, 19]. However, the evidence of an association between *Brucella* seropositivity and adverse pregnancy outcomes is still inconclusive from previous studies which may be due to the use of various serological tests such as the Rose Bengal Plate Test, tube agglutination or an enzyme-linked immunosorbent assay and the cross-sectional study was applied in most studies [8, 16, 20, 21]. To evaluate a causal relationship between brucellosis seropositivity and adverse pregnancy outcomes, a prospective study using serological diagnostic tool with high specificity for antibodies testing should be carried out.

Previous studies on the history of exposure to animals and/or raw animal products prior to or during pregnancy and the association with adverse pregnancy outcomes have focused on symptomatic rather than asymptomatic pregnant women [16, 17, 22]. The time from exposure to an infected animal to the detection of seropositivity in humans can vary from a week up to 10 years which can be indicated acute, chronic or previous infection [23]. Brucellosis can be diagnosed when the serological results are positive which is more common in symptomatic than asymptomatic individuals [23–25].

In Thailand, the rate of spontaneous abortion was 6.9% of total pregnancies [26], and rates of preterm and low birth weight newborns were 13.7% and 8.4%, respectively [27]. However, to date in Thailand there have been no studies examining brucellosis in pregnant women and to what extent it might be related to adverse pregnancy outcomes. Understanding the factors associated with exposure to animal or raw animal products and the relation of such exposure to the pregnancy risk of brucellosis would be useful for health education and promotion [28]. This study aimed (i) to assess the association of a history of exposure to goats and/or raw goat products and the serological status of anti-*Brucella abortus* immunoglobulin G with adverse pregnancy outcomes among pregnant women, and (ii) to explore factors associated with having exposure to goats and/or raw goat products among pregnant women.

## Methods

### Study design and setting

This prospective cohort study was conducted in 4 of the 16 districts of Songkhla province in southern Thailand, where meat and dairy goat production associated with the Thai Muslim communities is commonplace for household consumption and marketing. Songkhla province has been reported as the highest endemic area of animal brucellosis in southern Thailand and goats were identified to be the most infected animal during brucellosis outbreaks in this area [29, 30].

Four districts, Thepa, Chana, Saba Yoi, and Na Thawee, were chosen for the study because the registered number of households rearing goats were in the top five provincial rankings, accounting for up to 22% of all goats being raised [30]. Antenatal care (ANC) for pregnant women in the study district is provided in the Primary Care Units of a district hospitals and the Health Promoting Hospitals in the subdistrict level of each district. Information on ANC and delivery of pregnant women within the districts is routinely recorded and monitored by the district hospital.

### Participants

In Thailand, all pregnant women are encouraged to attend ANC which is provided by skilled attendants, with the first visit before 12 weeks of gestation as recommended by the World Health Organization (WHO). The number of ANC visits for low-risk pregnancies is at least 5 times: < 12, 16–20, 24–28, 30–34, and 36–40 weeks are the normal recommendation. For this study, pregnant Thai women aged 15–49 years who had a gestational age of 28 weeks or less coming for their first ANC visit at the study hospitals during July 2015–July 2016 and who planned to give birth at the responsible district hospital were included.

### Data collection

All eligible pregnant women presenting as noted above were informed about the study and invited to participate. After agreeing and signing the consent form, they were interviewed using a structured questionnaire and a blood sample for serological testing was taken. All women were followed until the completion of the study. The pregnancy outcomes were recorded by the health personnel who provided ANC.

### Tools and measurements

The structured questionnaire used in the study was divided into 4 main parts: socio-demographic and family characteristics, obstetric history, including previous history of one or more adverse pregnancy outcomes in prior pregnancies, types of home animals, and history of exposure to goats and raw goat products. The questionnaire was completed within 10–15 min by either a research assistant or the pregnant women themselves on the date of their first ANC visit.

Three-mL blood samples were collected in plain tubes separately from the ANC routine blood test and transported to the laboratories of the district hospitals. The serum was separated from the clotted blood samples and stored at − 20 °C. Due to cost restrictions, 500 test kits were prepared that these samples were sufficient to detect the proportion of positive titer at 5%. The serum samples to be tested were chosen by simple random sampling using computer-generated random numbers, and tested for anti-*Brucella abortus* immunoglobulin G (IgG) by enzyme-linked immunosorbent assay (ELISA) using a commercial ELISA anti-*Brucella abortus* IgG kit manufactured by EUROIMMUN (Lübeck, Germany) at the Immunology and Virology Unit laboratory at Songklanagarind Hospital, Songkhla province. The sensitivity and specificity of the kit were 78.0% and 98.0%, respectively. Following the recommendations of the manufacturer without adjustment, a value of 22 relative units/mL or more was considered as positive. This cut-off level of IgG from a single serum sample cannot differentiate between acute and chronic infection.

### Variables

#### Outcome measures

The occurrence of any adverse pregnancy outcomes defined as abortion, stillbirth, preterm birth and low birth weight newborn was the main outcome measure. Abortion was defined as premature expulsion of an embryo or fetus at gestational age of 23 weeks or less or weighing less than 500 g. Stillbirth was defined as birth of a baby showing no signs of life. Preterm birth was a birth before 37 completed gestational weeks and newborn weighing 2500 g or less was classified as low birth weight newborn.

#### Exposure status and independent variables

Having a history of any exposure to goats or raw goat products one or more times in their lives before the serology blood test at the first ANC visit was the criterion used for categorizing the pregnant women into unexposed and exposed groups. The exposure was then stratified by route of exposure as either cutaneous contact or consumption. Cutaneous contact was defined as a history of physical contact with a live goat or any of the various activities involved with rearing goats such as cleaning goat shelters, helping with goat births, or disposing goat carcasses, or otherwise having physical contact with raw goat meat or milk including slaughtering, meat cutting or milking. Consumption included a history of consumption of raw goat meat or milk.

Independent variables recorded were demographic characteristics, household environment factors related to animals, obstetric details of the current pregnancy, and previous history of adverse pregnancy outcomes. The demographic characteristics were age, religion, education attainment and occupation. Household environment factors related to animals included having home animals and types of home animals. Obstetric details of the current pregnancy were gestational age at first ANC, gravida, and parity. Previous history of adverse pregnancy outcomes defined as abortion, stillbirth, preterm birth and low birth weight newborn.

#### Sample size calculation

The comparison of two proportions formula was used for sample size calculation, and based on a literature review that reported the prevalences of adverse pregnancy outcomes among pregnant women with and without livestock exposure the required percentages were 20 and 10%, respectively [31]. Given a two-tailed alpha of 5% and a power of 80% to detect this difference, at least 169 exposed and 338 unexposed pregnant women (using a ratio of 1 to 2 as found from a pilot study) were needed. After 15% exclusion and assuming a 15% loss to follow-up rate and 10% non-response rate, a total of 663 women were required.

### Data analysis

All data were verified by double entry using EpiData version 3.1. Statistical analysis for both subgroups was performed with R version 3.5.0 and the Epicalc version 3.4.3.0 package. Distribution of exposure and adverse pregnancy outcomes were calculated descriptively in percentages. Associations between demographic characteristics, household environment, obstetric information, and exposure to goats and raw goat products and adverse pregnancy outcomes were analysed using univariate analysis and multiple logistic regression. A *p* value of 0.2 was used to include the factors in the regression analysis. A *p* value of 0.05 was set as the significance level.

## Results

**Fig. 1:**
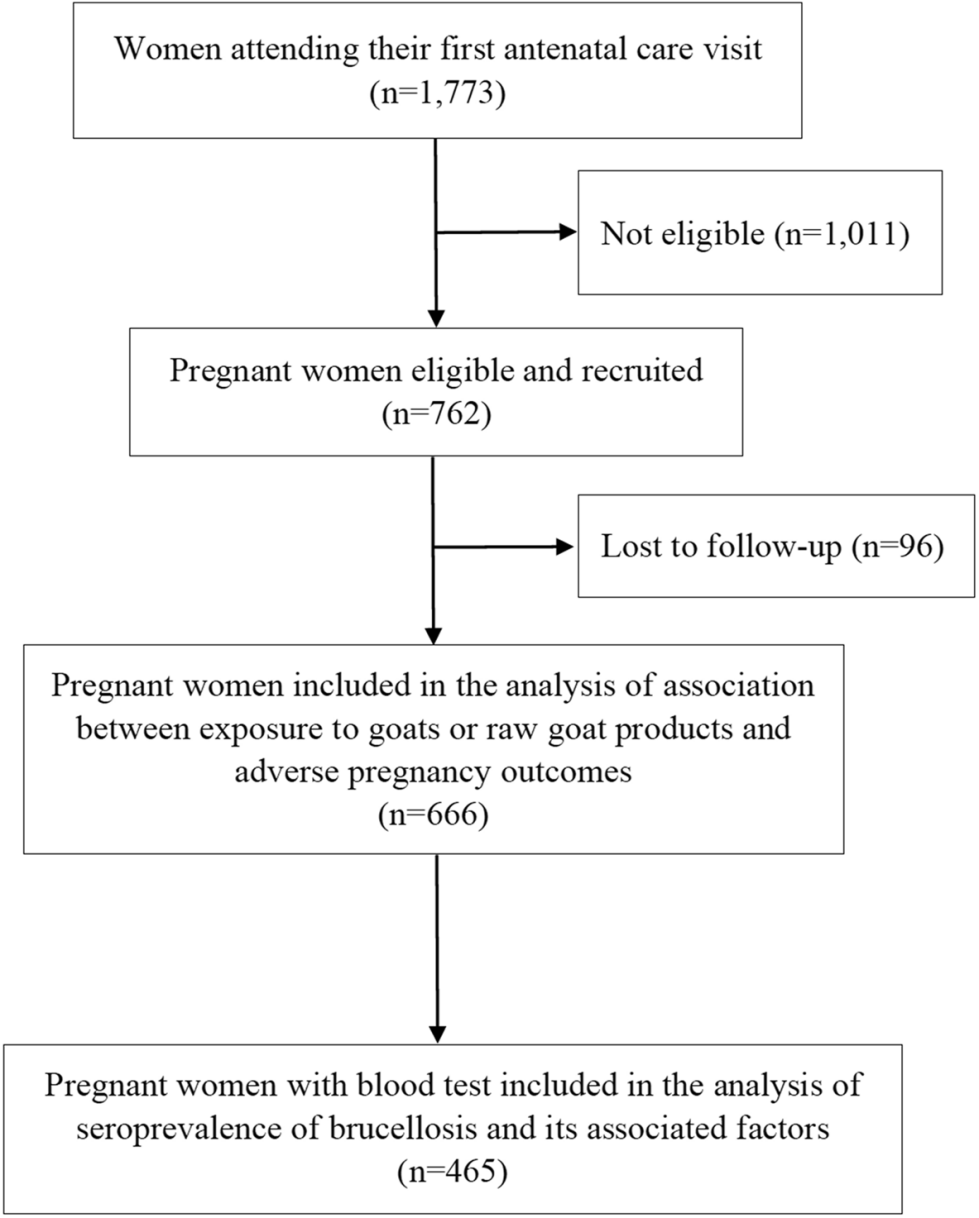
Flow diagram of the study

Of 1773 pregnant women attending their first ANC visit during the study period, 1011 women with more than 28 weeks of gestational age, who were non-Thai, who planned to give birth at a hospital other than the responsible district hospital, or unable to make decision to participate our study at date of first ANC visit were not eligible for the study, leaving a total of 762 women who fulfilled the criteria and were invited to participate in the study. All the women who were invited agreed to participate in the study. A flow diagram of the study is shown in Fig. 1. At the end of the follow-up period, 666 women were analysed for association between exposure status and occurrence of adverse pregnancy outcomes, and 465 for the brucellosis seroprevalence.

Table 1 presents the women’s demographic characteristics, obstetric information, and household environment information. The distributions of those characteristics and information among all women (*n* = 666) and women with blood tests (*n* = 465) were similar. The ages of the 666 women ranged from 15 to 44 years (mean ± sd. = 27.4 ± 6.3 years), with most aged 20–34 years. Most were Muslim, with education attainment of secondary school, agriculture workers and laborers and low personal monthly income. More than 80% of them had their first ANC visit at first trimester and one-fourth were primigravida. Three-fourths of women came for the first visit before 12 weeks of gestation as the national recommendation. One-fourth had a history of adverse pregnancy outcomes, of which abortion was the most common followed by preterm birth or low birth weight newborn and then stillbirth. Two-thirds of them had home animals and one-third reported that they had a neighbour rearing goats nearby their household.

**Table 1:** Demographic characteristics, obstetric information, and household environments of the study women

Characteristic	All pregnant women (*n* = 666)	Pregnant women with blood test (*n* = 465)	*p* value^*^
	n (%)	n (%)	
Demographic characteristics			
Age group, in years			0.956
15–19	74 (11.1)	51 (11.0)	
20–34	496 (74.5)	344 (73.9)	
35–49	96 (14.4)	70 (15.1)	
Religion			0.060
Muslim	594 (89.2)	431 (92.7)	
Buddhist or others	72 (10.8)	34 (7.3)	
Education			0.979
Primary school or lower	178 (26.7)	123 (26.5)	
Secondary school	375 (56.3)	261 (56.1)	
College/university	113 (17.0)	81 (17.4)	
Occupation			0.692
Agricultural worker or laborer	351 (52.7)	237 (50.9)	
Salesperson, teacher, clerk, or technician	164 (24.6)	125 (26.9)	
Housewife	151 (22.7)	103 (22.2)	
Personal monthly income, in USD			0.847
0–184	391 (58.7)	265 (57.0)	
185–368	213 (32.0)	155 (33.3)	
≥ 369	62 (9.3)	45 (9.7)	
Obstetric information			
Gestational age			0.418
1^st^ trimester (< 14 weeks)	560 (84.1)	400 (86.0)	
2^nd^ trimester (14–28 weeks)	106 (15.9)	65 (14.0)	
Gravida			0.909
Primi-gravida	178 (26.7)	119 (25.6)	
Multigravida	427 (64.1)	302 (64.9)	
Grand-multigravida	61 (9.2)	44 (9.5)	
Parity			0.976
Nulliparity	198 (29.7)	136 (29.2)	
Primiparity	241 (36.2)	171 (36.8)	
Multiparity	227 (34.1)	158 (34.0)	
Having history of abortion	103 (15.5)	81 (17.4)	0.381
Having history of stillbirth	11 (1.7)	7 (1.5)	0.847
Having history of preterm birth or low birth weight	84 (12.6)	66 (14.2)	0.441
Household environment			
Having cats, dogs, or rabbits	309 (46.4)	218 (46.9)	0.872
Having chickens or ducks	199 (29.9)	141 (30.3)	0.873
Having other birds	147 (22.1)	104 (22.4)	0.907
Having goats	103 (15.5)	92 (19.8)	0.059
Having cattle	20 (3.0)	19 (4.1)	0.326
Having neighbor rearing goats	243 (36.5)	172 (37)	0.863

^*^p value: chi-square test

Of 666 women, 204 (30.6%) had a history of any exposure to goats or raw goat products of which 64.7% of them were exposed via cutaneous contact and 36.8% of them were exposed by the consumption of raw goat products. Of the 465 women who had complete data of exposure and serum samples, 17 (3.7%) had positive serological results for anti-*Brucella abortus* IgG. Women who had positive serological results were more likely to a history of drinking raw goat milk than those who had negative results (6/17, 35.3% vs 42/448, 9.4%, *p* = 0.005), respectively.

One hundred and fifteen women (17.3%) had had one or more adverse pregnancy outcomes, which were abortion in 42 (42/115, 36.5%), stillbirth in 2 (2/115, 1.8%) and preterm birth or low birth weight in 71 (71/115, 61.7%). Table 2 shows factors associated with occurrence of any adverse pregnancy outcome among all pregnant women and those with blood tests. There was no significant association between exposure status and serological result for anti-*Brucella abortus* IgG with adverse pregnancy outcomes. Higher odds ratios of adverse pregnancy outcomes were found in women having their first ANC visit during the first trimester, a history of preterm and low birth weight newborns.

**Table 2:** Factors associated with occurrence of any adverse pregnancy outcome among all pregnant women (*n* = 666) and those with blood tests (*n* = 465)

Factor^a^	All pregnant women (*n* = 666) Adverse pregnancy outcome (*n* = 115)			Pregnant women with blood test (*n* = 465) Adverse pregnancy outcome (*n* = 112)		
	Crude OR (95% CI)	Adjusted OR^b^ (95% CI)	*p* value (LR test)	Crude OR (95% CI)	Adjusted OR^b^ (95% CI)	*p* value (LR test)
Demographic characteristic						
Age group, in years: ref. = 15–19			–			–
20–34	1.3 (0.6, 2.5)	–		1.2 (0.6, 2.5)	–	
35–49	1.0 (0.4, 2.3)	–		0.9 (0.4, 2.2)	–	
Religion: Buddhist or others vs Muslim	0.7 (0.3, 1.4)	–		1.2 (0.5, 2.5)	–	
Education: ref. = College/university or above			–			–
Primary school or lower	0.9 (0.5, 1.6)	–		0.8 (0.4, 1.6)	–	
Secondary school	0.9 (0.5, 1.6)	–		0.9 (0.5, 1.6)	–	
Occupation: ref. = Housewife			–			–
Agricultural worker or laborer	1.0 (0.6, 1.6)	–		0.9 (0.5, 1.6)	–	
Saleperson, teacher, clerk, or technician	1.1 (0.6, 2.0)	–		0.9 (0.5, 1.7)	–	
Personal monthly income, in USD: ref. = 0–184			–			–
185–368	0.8 (0.5, 1.3)	–		0.8 (0.5, 1.2)	–	
≥ 369	1.1 (0.6, 2.1)	–		1.1 (0.5, 2.2)	–	
Houseshold environment						
Having goats as home animal	1.2 (0.7, 2.0)	–	–	0.8 (0.5, 1.4)	–	–
Having other home animals excluding goats	1.4 (0.9, 2.1)	–	–	1.5 (1.0, 2.3)	–	–
Having neighbor rearing goats	1.1 (0.7, 1.7)	–	–	1.0 (0.7, 1.6)	–	–
Obstetric information						
Gestational age at first antenatal care visit: 1^st^ trimester (< 14 weeks) vs 2^nd^ trimester (14–28 weeks)	1.9 (1.1, 3.5)	2.0 (1.1, 3.7)	0.015	1. 7 (0.9, 3.1)	1.9 (1.0, 3.6)	0.041
Gravida: ref. = Primi-gravida			–			–
Multigravida	0.8 (0.5, 1.2)	–		0.7 (0.4, 1.1)	–	
Grand-multigravida	1.0 (0.5, 2.1)	–		0.8 (0.4, 1.8)	–	
Parity: ref. = Nulliparity			–			–
Primiparity	0.8 (0.5, 1.3)	–		0.7 (0.4, 1.2)	–	
Multiparity	1.0 (0.6, 1.6)	–		0.9 (0.5, 1.5)	–	
Having history of abortion	1.0 (0.6, 1.8)	–	–	0.8 (0.5, 1.5)	–	–
Having history of stillbirth	1.1 (0.2, 5.0)	–	–	1.3 (0.2, 6.6)	–	–
Having history of preterm birth	3.7 (1.7, 7.9)	2.4 (1.0, 5.7)	0.046	4.1 (1.7, 9.8)	3.6 (1.4, 9.3)	0.010
Having history of low birth weight	3.1 (1.7, 5.6)	2.6 (1.3, 4.9)	0.007	2.7 (1.4, 5.1)	2.2 (1.1, 4.3)	0.037
Exposure status – cutaneous contact						
Live goats or goat rearing activities	1.4 (0.9, 2.2)	–	–	0.8 (0.5, 1.3)	–	–
Raw goat meat	0.7 (0.3, 1.6)	–	–	0.5 (0.2, 1.1)	0.4 (0.2, 1.0)	0.035
Raw goat milk	0.3 (0.0, 2.2)	–	–	0.2 (0.0, 1.6)	–	–
Exposure status – consumption						
Raw goat meat	0.8 (0.3, 2.2)	–	–	0.6 (0.2, 1.6)	–	–
Raw goat milk	0.6 (0.3, 1.5)	–	–	0.4 (0.2, 1.0)	–	–
Serological result for anti*-Brucella abortus IgG*						
Positive vs negative	–	–	–	0.2 (0.0, 2.0)	0.2 (0.0, 1.4)	0.041

^a^yes vs no where reference level is no

^b^adjusted by backward stepwise method; *OR* odds ratios, *CI* confidence interval of odds ratios, *LR-test* likelihood ratio test

High personal monthly income and history of abortion were associated with consumption of raw goat products, but they were found to be not significant in multiple logistic regression. The factors associated with exposure from cutaneous contacts are presented in Table 3. Women having goats as a home animal were more likely to be exposed to goats or raw goat products by cutaneous contact with an adjusted odds ratio of 15.2 (95% CI 9.8–26.1) and the adjusted odds ratio of women having a neighbor rearing goats was 2.0 (95% CI 1.3–3.0).

**Table 3:** Factors associated with having personal exposure from cutaneous contact among pregnant women (*n* = 666)

Factor^a^	Having exposure from cutaneous contact		
	Crude OR (95%CI)	Adjusted OR (95%CI)	*p* value (LR-test)
Religion: Buddhist and others vs Muslim	0.4 (0.2, 0.8)	0.6 (0.3, 1.3)	0.184
Having goats as home animals	14.8 (9.1, 24.1)	15.2 (8.9, 26.1)	< 0.001
Having other home animals excluding goats	0.7 (0.5, 1.0)	1.5 (0.9, 2.3)	0.100
Having neighbor rearing goats	2.7 (1.9, 3.9)	2.0 (1.3, 3.0)	0.002

*OR* odds ratio, *CI* confidence interval of odds ratios, *LR-test* likelihood ratio test

^a^yes vs no where reference level is no

## Discussion

No evidence of association of past exposure to goats and raw goat products and the serological status of anti-*Brucella abortus* IgG with adverse pregnancy outcomes was found; however, a higher rate of seropositivity was found among pregnant women who had been exposed to goats or raw goat products. A history of previous preterm or low birth weight newborns were independent risk factors for adverse pregnancy outcomes. Women having goats as home animals and having a neighbour rearing goats were more likely to be exposed to goats and raw goat products by cutaneous contact.

No association was found between exposure to goats and raw goat products with adverse pregnancy outcomes in our study, which was similar to the findings of a large cohort study from Denmark, although the study designs and methodologies were different. Compared to our study, the Denmark study focused on a wide variety of animals rather than goats only, and short duration for notification of exposure collected and the study reported lower rate of cutaneous contact [32]. Although approximately one-third of the women in our study said they had been exposed to goats and raw goat products at least one time before this current pregnancy, they were less likely to be exposed during pregnancy as also found in previous studies [33–35]. According to this, the time point that we asked for retrospective women’s exposure to goats and raw goat products and serum testing for IgG detection was able to explain the association with their adverse pregnancy outcomes.

No relationship between seropositivity and adverse pregnancy outcomes in our study may be explained by the study women were normal pregnant women and IgG detection representing possibly past infection in women’s life. Our finding was different from the previous studies that adverse pregnancy outcomes were associated with acute infection in symptomatic pregnant women study [8, 36]. Women with a previous preterm or low birth weight newborn had an increased risk of adverse pregnancy outcomes in their current pregnancies, as other studies have reported the same tendency [37–39].

Goat ownership and neighbors nearby rearing goats were associated with cutaneous contact with live goats or raw goat products among the study women due to the fact that animal owners are more likely to have physical contact with their own animals or the rearing environment [35, 40]. Moreover, women have more opportunity to be exposed to goats rather than other larger domestic livestock animals such as cattle or water buffalos. Goat rearing in nomadic conditions in community environments is observed in rural areas of Bangladesh, Nepal and Thailand, including our study setting in which small-scale animal production farms with poor management are common, regardless of goat ownership [34].

Exposure to goats and raw goat products was emphasized as the main interest of our study due to its influence on disease transmission and negative health consequences. Zoonotic infections which have been shown to be transmitted to pregnant women in endemic areas in previous studies include toxoplasmosis, brucellosis, and coxiellosis [7, 11]. *Brucella abortus* is one of the common pathogens causing human brucellosis which can be found in various home animals such as cattle, goats and sheep [15, 41–43]. Our study found higher detection of anti-*Brucella abortus* IgG among pregnant women who were exposed to goats and raw goat products, as was also reported in studies conducted in Jordan and Afghanistan which focused on contact with unpasteurized dairy products or goat milk [11, 13]. The low seroprevalence of brucellosis among pregnant women in our study was slightly lower than studies from Yemen [44] and Pakistan [20] in which the study participants were asymptomatic pregnant women. However, our finding was lower when compared with studies conducted among slaughterhouse workers in Pakistan (21.7%) and goat farmers and livestock officers in Thailand (8.3–8.8%) [45, 46].

One-fourth of the women in our study reported cutaneous contact, mainly from live goats or while doing goat-rearing activities. This was likely related to ritual Islamic activities as the majority of our participants were Muslim, such as “Aqeeqah”, the ritual animal sacrifice on the occasion of a new birth, and “Qurbani”, the Islamic religious practice of an animal sacrifice offering to Allah [47]. Good hand hygiene practicing among people having extensive exposure to animal raising or contaminated environments and avoiding food-borne zoonosis by cooking meat well or boiling milk before consuming them should be promoted [32, 48]. For women, an explanation of the dangers involved with being around goats can be explained during ANC visits; however, a previous study found a low rate of healthcare providers gave information about hand or food hygiene for their patients who exposed to animals [49].

There were some limitations to our cohort study measuring the exposure of goats and raw goat products and seropositivity of brucellosis among pregnant women in southern Thailand. First, a history of exposure in our study included at any time before the date of the first ANC visit for the current pregnancy, and thus recall bias may have occurred; however, these exposure events were distinctive for women. Second, only past history of infection could be diagnosed and some acute or chronic infections may have been missed because only a single serum sample was taken from each woman and the ELISA IgG test was done retrospectively thus a second sample to provide a pair of positive samples could not be tested. Third, data on smoking or drinking were not collected due to the very low rate of these habits in pregnant women in the south of Thailand. Forth, the exposure was measured cross-sectionally at one time only, during the first ANC visit, and was not rechecked during the remaining pregnancy, which may have affected the true association between exposure and adverse pregnancy outcome. Finally, women who were pregnant and aborted before coming for an ANC visit could have been missed from our study, which could have affected the results.

## Conclusion

Our study found no association between past exposure with goats and/or raw goat products and adverse pregnancy outcomes, but such exposure was significantly associated with brucellosis seropositivity. Effective counseling on avoiding consumption of raw goat milk would be beneficial to prevent goat-related infection in pregnant women in our study setting.

## Abbreviations

ANC: Antenatal care
CI: Confidence interval
ELISA: Enzyme-linked immunosorbent assay
IgG: Immunoglobulin G
LR: Likelihood ratio
mL: Milliliter
OR: Odds ratios
USD: United States dollar
WHO: World Health Organization
